# Innate immune regulation of adaptive immunity: mechanisms, implications, and bias

**DOI:** 10.3389/fimmu.2026.1847470

**Published:** 2026-05-28

**Authors:** Yikai Zhang, Yuao Qin, Zekun Cheng

**Affiliations:** Third Xiangya Hospital, Central South University, Changsha, China

**Keywords:** adaptive immunity, allergic diseases, autoimmunity, immune dysregulation, infection, innate immunity

## Abstract

Innate immunity is not merely an early defensive system but a key regulator of adaptive immune fate. Through pattern-recognition receptor signaling, antigen presentation, cytokine production, and metabolic–epigenetic reprogramming, innate immune responses shape the strength, duration, and direction of T- and B-cell immunity. This review summarizes how innate immune regulation of adaptive immunity contributes to immune dysregulation in infection, autoimmunity, and allergic disease. We focus on three major mechanisms: remodeling of antigen presentation and costimulation, reshaping of cytokine microenvironments that guide T helper cell polarization, and metabolic–epigenetic programming associated with trained immunity or immune tolerance. We further propose that disease outcomes can be interpreted through three regulatory dimensions of innate immune signaling: insufficient signal strength promotes defective pathogen control and weak adaptive priming; persistent or excessive activation sustains autoimmune inflammation and loss of tolerance; and type 2–biased epithelial–innate signaling drives allergic inflammation through the alarmin–ILC2–Th2–IgE axis. By integrating molecular signaling, innate immune cell crosstalk, metabolic regulation, and epigenetic remodeling, this review provides a concise framework for understanding how innate immune imbalance shapes adaptive immune dysfunction and highlights therapeutic opportunities targeting interferon pathways, inflammasomes, epithelial alarmins, metabolic programs, and microbiome-related immune regulation.

## 1 Introduction

### 1.1 Innate and adaptive immunity: from division to regulation

The immune system is traditionally divided into innate and adaptive immunity. Innate immunity provides rapid host defense through epithelial barriers, pattern-recognition receptors (PRRs), phagocytes, dendritic cells (DCs), natural killer (NK) cells, and innate lymphoid cells (ILCs), whereas adaptive immunity is mediated mainly by antigen-specific T and B cells and is characterized by clonal expansion and immune memory. Classic studies established that PRRs recognize conserved microbial or danger-associated molecular patterns and initiate inflammatory programs that help distinguish infectious non-self from self (1).

This division, however, is increasingly understood as functional rather than independent. Innate immune activation not only initiates host defense but also instructs adaptive immune fate. For example, Toll-like receptor signaling promotes DC maturation and thereby contributes to the initiation and shaping of adaptive immune responses (2). Through antigen presentation, costimulatory signaling, and cytokine production, innate immune cells influence T-cell priming, helper T-cell polarization, B-cell activation, and immune tolerance.

Therefore, innate immunity can be viewed as an upstream regulatory layer of adaptive immunity. Its impact depends not simply on whether it is activated, but on the strength, duration, and qualitative bias of the signal. Defective signaling may impair pathogen control, persistent activation may promote loss of tolerance, and type 2–biased epithelial–innate signaling may drive allergic inflammation.

### 1.2 Emerging concepts: trained immunity, barrier immunity, and immune-state programming

Recent advances have further expanded the concept of innate immune regulation. Trained immunity describes long-lasting functional changes in innate immune cells after microbial or inflammatory stimulation, driven largely by metabolic rewiring and epigenetic remodeling rather than antigen-specific receptor rearrangement (3). This provides a mechanism by which previous innate stimulation can alter later cytokine production, antigen-presenting capacity, and downstream adaptive responses.

Barrier immunity has also emerged as a central regulator of adaptive immune outcomes. Epithelial tissues in the skin, airways, and gut not only limit antigen entry but also release mediators that shape local immune responses. In allergic disease, epithelial-derived alarmins such as TSLP, IL-33, and IL-25 activate ILC2s and condition DCs, thereby promoting type 2 immunity and Th2-associated responses (4, 5). Similarly, microbial signals and metabolites at mucosal sites contribute to immune tolerance and Treg/Th17 balance.

These findings support the idea that innate immunity sets the baseline state of adaptive immune responsiveness. Genetic susceptibility, metabolic disturbance, microbiome dysbiosis, barrier disruption, aging, and prior immune exposure can each modify innate immune thresholds and thereby influence whether adaptive immunity becomes protective, tolerogenic, exhausted, or pathogenic.

### 1.3 Scope of this review

This review focuses on how innate immune changes regulate adaptive immune dysfunction in three disease contexts: infection, autoimmunity, and allergic disease. We organize the discussion around three major mechanisms: antigen presentation and costimulatory remodeling, cytokine microenvironment formation, and metabolic–epigenetic reprogramming.

We further use signal strength, duration, and bias as an integrated framework. In this model, insufficient innate immune signaling contributes to defective pathogen clearance and weak adaptive priming; persistent or excessive activation promotes loss of tolerance and autoimmune feedback loops; and type 2–biased epithelial–innate signaling drives allergic inflammation through the alarmin–ILC2–Th2–IgE axis. This framework is used to connect upstream modulatory factors, innate immune cell subsets, adaptive immune consequences, and potential therapeutic strategies.

To avoid using these dimensions only as conceptual labels, we define them operationally throughout the review by linking them to measurable pathway features. Signal strength is reflected by the amplitude of PRR-driven NF-κB/IRF activation, cytokine production, antigen presentation, and costimulatory molecule expression. Signal duration is reflected by the kinetics and persistence of pathway activation, such as transient versus sustained IFN-stimulated gene expression or inflammasome activity. Signal bias refers to the dominant qualitative output of innate signaling, including IFN-I-dominant, IL-1β/IL-18-dominant, IL-12-dominant, tolerogenic, or epithelial alarmin-dominant programs that differentially guide adaptive immune outcomes.

## 2 Core mechanisms by which innate immunity regulates adaptive immunity

### 2.1 Barrier immunity as the first regulatory interface

Barrier tissues constitute the first interface between environmental signals and the immune system. The skin, airway, and intestinal epithelium restrict the entry of pathogens, allergens, and irritants through tight junctions, mucus production, antimicrobial peptides, and coordinated tissue-repair programs. However, barrier immunity is not only defensive; it also regulates the threshold and quality of downstream immune activation (6, 7).

Epithelial cells act as immune sentinels by sensing microbial products, tissue damage, and environmental stress, and by releasing mediators that shape local innate and adaptive responses. In mucosal tissues, epithelial-derived cytokines, chemokines, antimicrobial peptides, and metabolites influence antigen sampling, DC activation, ILC responses, and T-cell differentiation. Thus, barrier integrity helps determine whether antigen exposure promotes tolerance, protective immunity, or inflammation (8).

This regulatory role is especially evident in allergic disease. When epithelial barriers are damaged, alarmins such as thymic stromal lymphopoietin (TSLP), IL-33, and IL-25 are released and activate ILC2s and DCs, thereby promoting type 2 immune responses and Th2 polarization (4, 5). In the gut, microbial ligands and metabolites contribute to mucosal immune tolerance and help maintain the balance between regulatory T cells and inflammatory T-cell subsets. Therefore, barrier immunity should be viewed as an upstream organizer of innate-adaptive immune crosstalk rather than a passive physical shield.

### 2.2 Pattern-recognition receptors and signaling modulation

Pattern-recognition receptors are central sensors of innate immunity. PRRs recognize pathogen-associated molecular patterns and damage-associated molecular patterns, initiating signaling cascades that induce inflammatory cytokines, type I interferons, antimicrobial programs, and antigen-presenting functions. Classical work established that innate immune recognition relies on germline-encoded receptors that distinguish infectious non-self from noninfectious self (9).

**Figure 1:**
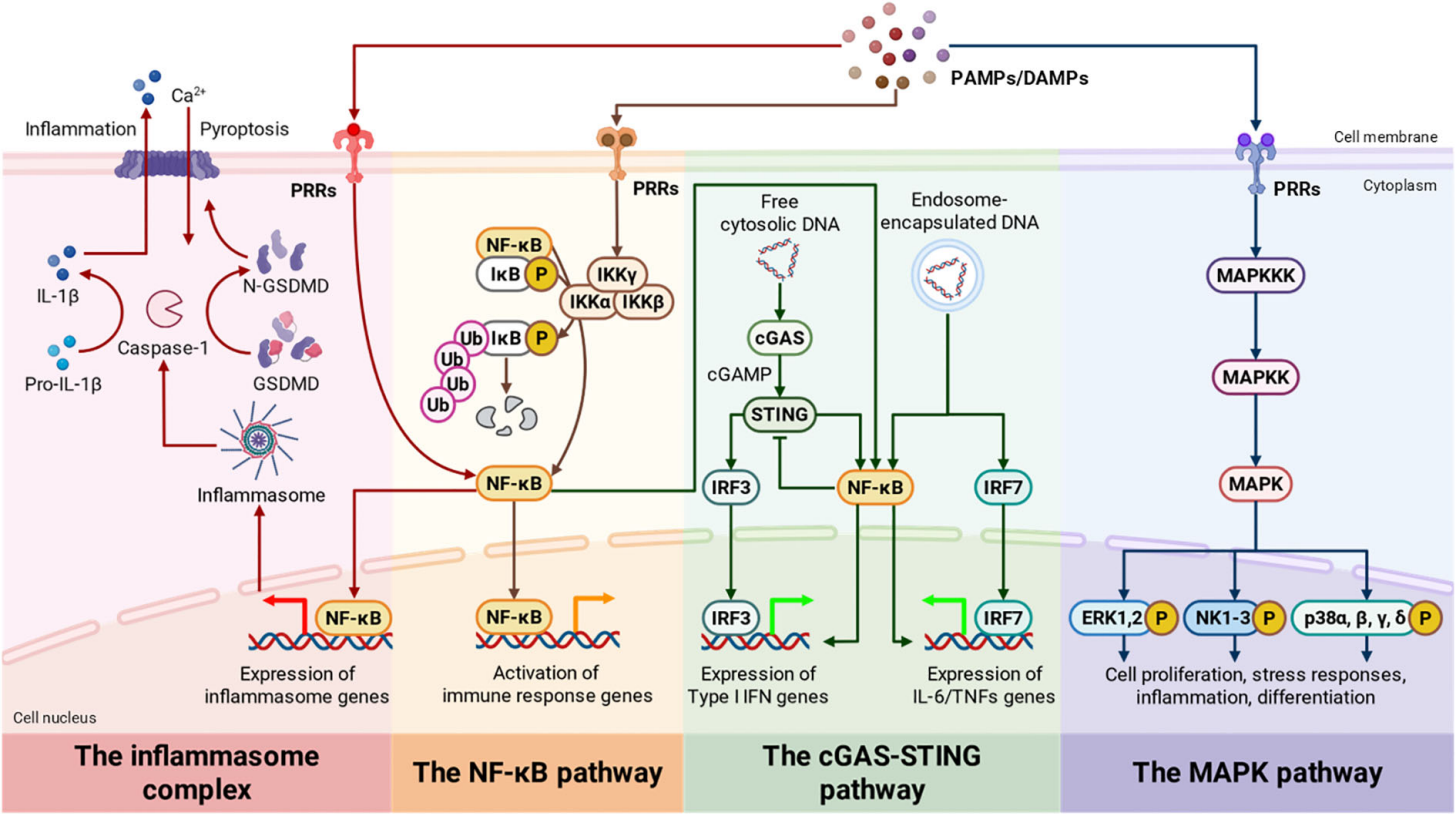
Overview of innate immune signaling pathways. This diagram illustrates the activation of four key innate immune signaling pathways: the inflammasome pathway, mediating inflammation and pyroptosis through Caspase-1 and N-GSDMD; the NF-κB pathway, activated by PRRs to regulate immune gene expression; the cGAS-STING pathway, which senses cytosolic DNA and promotes type I interferon and inflammatory cytokine production; and the MAPK pathway, controlling cell proliferation, stress response, and immune functions through a cascade.

TLRs, NLRs, RLRs, and cGAS-STING represent major PRR systems. TLRs activate downstream pathways such as NF-κB and IRFs, leading to inflammatory and antiviral responses. NLRs, particularly NLRP3, participate in inflammasome assembly and IL-1β/IL-18 maturation (10). RLRs detect viral RNA and induce antiviral signaling, whereas cGAS-STING senses cytosolic DNA and drives type I interferon production. These pathways converge on shared transcriptional and inflammatory programs but differ in cellular localization, ligand specificity, and disease relevance (**Figure 1**).

For adaptive immunity, the key point is that PRR signaling does not simply “activate” immunity in an on/off manner. Instead, it tunes the strength, duration, and bias of immune responses. PRR signaling can determine the degree of DC maturation, the level of MHC and costimulatory molecule expression, and the cytokine profile that guides T-cell differentiation. TLR signaling is particularly important in linking innate recognition to adaptive immune activation, in part by inducing DC maturation and enabling efficient T-cell priming (10, 11).

Therefore, PRR pathways should be interpreted as regulatory circuits. Signal amplitude determines whether adaptive priming is weak or robust; signal persistence determines whether inflammation resolves or becomes chronic; and pathway bias, such as IFN-dominant, inflammasome-dominant, or type 2 alarmin–dominant signaling, influences the direction of adaptive immune polarization.

These dimensions can be illustrated by PRR signaling dynamics. For example, the strength of TLR or cGAS–STING activation influences the extent of NF-κB/IRF activation, type I interferon production, and DC maturation, thereby determining whether antigen presentation and T-cell priming are weak, sufficient, or excessive. Signal duration is reflected by whether PRR-induced transcriptional programs are rapidly resolved or persist as chronic IFN-I, inflammasome, or inflammatory cytokine activity. Signal bias arises when one output module dominates over others; IL-12-rich innate activation favors Th1 differentiation, IL-1β/IL-6/IL-23-skewed responses support Th17 inflammation, whereas epithelial TSLP/IL-33/IL-25-dominant signaling promotes ILC2 activation, Th2 polarization, IgE class switching, and allergic inflammation.

### 2.3 Innate immune cell subsets bridging innate and adaptive immunity

Innate-adaptive immune crosstalk is executed by specialized immune cell subsets rather than by soluble signals alone. DCs, monocytes/macrophages, neutrophils, NK cells, and ILCs each contribute distinct forms of information to adaptive immunity, including antigen availability, costimulatory signals, inflammatory tone, tissue context, and cytokine bias. A systematic view of these cell subsets helps clarify how innate sensing is translated into adaptive immune outcomes.

#### 2.3.1 Dendritic cells: antigen presentation, costimulation, and T-cell fate instruction

DCs are the major professional antigen-presenting cells linking innate recognition to adaptive immunity. After sensing pathogens or tissue damage, DCs capture and process antigens, upregulate MHC molecules and costimulatory ligands such as CD80, CD86, and CD40, and migrate to lymphoid tissues to prime naïve T cells. The importance of TLR-induced DC maturation in adaptive immune activation has been well established (12).

Beyond antigen presentation, DCs instruct T-cell fate through cytokine production and tissue-specific conditioning. Different DC subsets or activation states can favor distinct helper T-cell programs, including Th1, Th2, Th17, Tfh, and Treg differentiation. Contemporary reviews emphasize that DC subset identity, maturation status, and cytokine output are major determinants of CD4^+^ T helper cell differentiation outcomes (13). Therefore, DCs are not merely carriers of antigen but interpreters of innate signals that convert local danger cues into adaptive immune direction (14).

#### 2.3.2 Monocytes/macrophages and neutrophils: phagocytosis, inflammatory amplification, and antigen availability

Monocytes and macrophages participate in pathogen clearance, tissue repair, cytokine production, antigen presentation, and trained immunity. Their activation state influences whether adaptive immunity develops in a pro-inflammatory, regulatory, or tolerized direction. In inflammatory tissues, macrophage-derived cytokines such as IL-1β, IL-6, TNF, IL-12, and IL-23 can shape T-cell differentiation and sustain local immune responses (15, 16).

Macrophages are also central to metabolic–epigenetic immune programming. Trained macrophages can show enhanced cytokine responses after prior stimulation, whereas tolerized or exhausted macrophages may display reduced antimicrobial and antigen-presenting capacity. These states are linked to metabolic rewiring and epigenetic remodeling and provide a mechanism by which innate immune history influences later adaptive responses (14).

Neutrophils are traditionally viewed as short-lived effector cells, but they also influence adaptive immunity by shaping inflammatory environments and antigen availability. Through degranulation, reactive oxygen species, cytokine release, and neutrophil extracellular traps (NETs), neutrophils can amplify tissue inflammation and expose immunostimulatory material. In infection, these functions support early pathogen control; in autoimmunity, excessive NET formation may expand the self-antigen pool and promote pathogenic adaptive responses (17).

#### 2.3.3 NK cells and ILCs: early cytokine shaping and tissue-specific immune bias

NK cells provide early defense against virus-infected and transformed cells through cytotoxicity and cytokine production. Their production of IFN-γ can promote Th1-biased immunity and support macrophage activation, thereby linking early innate cytotoxic responses to later adaptive cellular immunity (18, 19).

ILCs are particularly important at barrier sites because they rapidly produce cytokines that resemble helper T-cell effector programs but do not require antigen-specific receptors. ILC2s produce IL-5 and IL-13 in response to epithelial alarmins and promote eosinophilic inflammation, mucus production, and type 2 immune bias. ILC3s produce IL-17 and IL-22, contributing to barrier defense and inflammatory regulation. In this way, ILCs help establish tissue-specific cytokine environments that can precede and reinforce adaptive T-cell polarization (17, 20).

### 2.4 Three core routes of innate-to-adaptive immune regulation

Although innate immunity influences adaptive immunity through many molecules and cell types, these effects can be organized into three major routes: antigen presentation and costimulation, cytokine microenvironment formation, and metabolic–epigenetic reprogramming (21). These three routes provide the mechanistic backbone of this review.

#### 2.4.1 Antigen presentation and costimulatory remodeling

Antigen presentation is the most direct route by which innate immune cells initiate adaptive immunity. DCs and macrophages process antigens and present them through MHC molecules to T cells, while costimulatory signals determine whether T cells become activated, anergic, regulatory, or memory-prone. Insufficient antigen presentation or weak costimulation may impair protective immunity, whereas excessive or misdirected presentation of self-antigens may support autoimmunity (22).

Thus, antigen presentation is not only a delivery system for antigenic information; it is a checkpoint that integrates PRR activation, tissue context, cytokine signals, and costimulatory strength to determine adaptive immune fate.

#### 2.4.2 Cytokine microenvironment and helper T-cell polarization

The cytokine microenvironment generated by innate immune cells is a major determinant of T-cell differentiation. IL-12 and IFN-γ favor Th1 responses; IL-4 and IL-13 support Th2 immunity; IL-6, IL-1β, TGF-β, and IL-23 contribute to Th17 differentiation; and TGF-β with IL-2 promotes regulatory T-cell development. Cytokines also influence Tfh differentiation, B-cell help, antibody class switching, and memory formation. Reviews of CD4^+^ T-cell differentiation emphasize that cytokines produced by DCs and other cells during priming are central determinants of helper T-cell fate (23, 24).

In disease, cytokine bias provides a mechanistic explanation for divergent adaptive outcomes. Weak inflammatory cytokine production may lead to poor T-cell priming in infection; persistent IFN-I and inflammatory cytokines may sustain autoimmunity; and epithelial alarmin-driven IL-4/IL-5/IL-13 environments promote allergic type 2 immunity.

#### 2.4.3 Metabolic–epigenetic reprogramming and trained immunity/tolerance

Innate immune cells can acquire persistent functional states through metabolic and epigenetic remodeling. In trained immunity, previous stimulation induces durable changes in chromatin accessibility, histone modifications, and metabolic pathways, resulting in enhanced responses to later challenges. In contrast, persistent or overwhelming stimulation may induce tolerance or immune paralysis, characterized by reduced cytokine production, impaired antigen presentation, and defective pathogen control (25).

This mechanism is important because it links past immune exposures to future adaptive immune outcomes. Metabolic pathways such as glycolysis, tricarboxylic acid cycle activity, mitochondrial function, and lipid metabolism can influence the availability of substrates or cofactors for chromatin-modifying enzymes. As a result, innate immune cells may become programmed toward hyper-responsiveness or hypo-responsiveness, thereby altering the threshold for adaptive immune activation. Trained immunity has been defined as an innate immune memory program mediated by epigenetic and metabolic reprogramming, with both protective and pathogenic implications (25).

In BCG-vaccinated human cohorts, trained immunity has been associated with sustained transcriptional changes in the hematopoietic progenitor compartment and enhanced myeloid responsiveness, providing an *in vivo* human context for long-term innate immune reprogramming. In β-glucan-trained human monocyte/macrophage models, enhanced secondary cytokine production has been linked to histone modification and metabolic rewiring. These findings support the interpretation that trained immunity influences adaptive immunity mainly in an indirect manner: trained myeloid cells may reshape the inflammatory context, antigen-presenting capacity, and cytokine signals that regulate T-cell priming and differentiation (26, 27).

Therefore, metabolic–epigenetic reprogramming provides a long-term regulatory layer beyond acute cytokine signaling. It helps explain why prior infection, vaccination, metabolic disease, aging, or chronic inflammation can durably alter immune responsiveness and disease susceptibility (**Table 1**).

**Table 1:** Epigenetic modifications on trained immunity.

Classification	Sites/types	Function	References
DNA methylation	5-mC	Classic DNA methylation marks show changes in methylation levels at promoters/enhancer regions of inflammatory genes during trained immunity, which may affect the stability of long-term memory.	(97, 98)
	5-hmC 5-fC 5-caC	TET enzyme-mediated methylation oxidation derivatives are sometimes associated with active enhancers/gene activation.	
Histone methylation	H3K4me3	Marks active promoters and is closely associated with enhanced expression of inflammatory genes; commonly seen after β-glucan/BCG training	(99, 100)
	H3K4me1	Often found in enhancer regions, it has a ‘memory imprint’ effect; it can be retained in a silent state even after training.	
	H3K9me2/H3K9me3	It is generally an inhibitory mark, which may decrease during training-induced immunity, thereby promoting an open chromatin state.	
Histone acetylation	H3K27ac	Active enhancer/promoter marks that open chromatin and promote transcription; often persist after training induction	(99, 101)
	Others (such as H3/H4 acetylation)	Enhance overall chromatin accessibility and promote gene expression	
Chromatin remodeling	chromatin accessibility	After training, chromatin becomes more accessible to transcription factors, which is an important reflection of specific epigenetic state changes.	(100)
Non-coding RNA regulation	microRNA/lncRNA	As part of the epigenetic mechanism, by regulating epigenetic enzymes (such as histone methyltransferases and demethylases) or directly affecting transcription	(100)
Histone-modifying enzymes affect	HMT (Set7/SETD7)	Write in active marks (such as promoting H3K4me1/3 deposition) to enhance the accessibility of inflammatory genes.	(100)
	KDM (KDM5)	Erase inhibitory methyl marks to maintain a training memory state	
	HAT/HDAC	Regulate acetylation levels, affecting chromatin accessibility and gene expression patterns	

## 3 Upstream modulators of innate immune states

Innate immunity is not a fixed defensive system. Its activation threshold, inflammatory tone, and response direction are shaped by genetic background, metabolic status, microbiome composition, barrier integrity, age, and previous immune exposures. These factors do not act independently; rather, they reshape innate immune signal strength, duration, and bias, thereby influencing how adaptive immunity is initiated, polarized, maintained, or suppressed. Understanding these upstream modulators is essential for linking innate immune changes to infection susceptibility, autoimmunity, and allergic disease (**Table 2**).

**Table 2:** Main factors affecting innate immunity.

Classification	Factor	Pathways/mechanisms
Genetic/Pathway Susceptibility	PRR gene mutation	TLR, NLR, RLR Signal pathway
			Nucleic acid recognition axis abnormality	cGAS–STING, RIG-I/IFIH1
	Related signal transduction elements	NF-κB, IRFs, MAPK
	Metabolic state	Energy metabolism Changes	Glycolysis, TCA, lipid metabolism
Metabolic disease		Obese/High-fat/Diabetic condition
			Mitochondrial dysfunction	Mitochondrial DNA release
Microbiome		Mucosal microbiota composition	PRR - Microbial Interactions
	Microbial metabolites	SCFAs, secondary bile acids, etc.
	Antibiotics/Diet	Microbiota disturbance
Barrier and Environmental Exposure	Epithelial integrity	Barrier Break
			Pollutants/Irritants	Smoke/Particulate Matter
	Allergen exposure	Pollen/Dust Mites
	Other factors	Age	Immunosenescence/Increased inflammatory baseline
History of Immune Training				BCG, vaccine exposure
Medication/Nutrition		Corticosteroids, vitamins, etc.

### 3.1 Genetic and pathway susceptibility: PRR–IFN axis and nucleic-acid sensing

Genetic variation in innate immune receptors and downstream signaling components can alter the threshold and magnitude of immune activation. Variants affecting PRR signaling, nucleic-acid sensing, or type I interferon (IFN-I) responses may change how efficiently the host detects pathogens or self-derived danger signals. This makes the PRR–IFN axis a key determinant of both antiviral protection and autoimmune risk. IFN-I responses are essential for antiviral defense, but excessive or persistent IFN-I activity is closely associated with systemic autoimmune diseases such as systemic lupus erythematosus (SLE) and Sjögren’s syndrome (28, 29).

Nucleic-acid sensing is particularly important because it sits at the boundary between host defense and autoimmunity. Endosomal TLR7/8/9 and cytosolic DNA-sensing pathways such as cGAS-STING are designed to detect viral or microbial nucleic acids; however, when self-DNA, self-RNA, mitochondrial DNA, or immune complexes accumulate, these same pathways can drive chronic innate activation. A well-known example is TLR7 gain-of-function, which has been shown to cause a lupus-like autoimmune phenotype in humans (30).

Thus, genetic or pathway-level susceptibility can push innate immunity in opposite directions depending on context. Reduced PRR–IFN signaling may weaken early antiviral control and impair adaptive priming, whereas excessive nucleic-acid sensing may generate persistent IFN-I signatures, enhance DC activation, and promote autoreactive T- and B-cell responses.

### 3.2 Metabolic status: obesity, hyperglycemia, lipid metabolism, and myeloid reprogramming

Metabolic status is a major regulator of innate immune function. Obesity, hyperglycemia, and lipid metabolic disturbance are associated with chronic low-grade inflammation and altered myeloid cell activation. In adipose tissue and metabolically stressed organs, macrophages and monocytes can acquire pro-inflammatory phenotypes that produce TNF, IL-1β, IL-6, and other mediators, thereby increasing systemic inflammatory tone.

Metabolic disturbance can also induce trained immunity-like programs. A representative experimental context comes from hyperglycemia-exposed macrophages and their precursors, in which high-glucose conditions induce trained immunity-like inflammatory memory and promote atherosclerosis. This provides a disease-relevant example showing that metabolic stress can imprint innate immune responsiveness through metabolic and epigenetic mechanisms, thereby altering the cytokine and antigen-presenting environment in which adaptive immune responses develop (31). Hyperglycemia has been shown to promote inflammatory memory in macrophages through metabolic and epigenetic reprogramming, providing a mechanism by which metabolic disease may produce sustained innate immune activation even after acute stimulation has resolved (32). Saturated fatty acids and lipid-derived metabolites can further modulate innate immune cells, linking diet and lipid metabolism to inflammatory set-points and disease susceptibility.

These metabolic effects are relevant to adaptive immunity because they reshape the cytokine milieu and antigen-presenting state of myeloid cells. A metabolically trained pro-inflammatory state may promote Th1 or Th17-skewed inflammation and contribute to autoimmune or chronic inflammatory disease, whereas severe or chronic metabolic stress may impair antimicrobial function and increase infection risk. Thus, metabolic status modifies both the intensity and persistence of innate immune signals.

### 3.3 Microbiome: mucosal homeostasis, immune tolerance, and Treg/Th17 balance

The microbiome is a major environmental regulator of innate and adaptive immunity, particularly at mucosal surfaces. Commensal microbes provide continuous low-level stimulation to epithelial cells and innate immune receptors, helping maintain barrier integrity, antimicrobial defense, and immune tolerance. Rather than simply activating immunity, microbial signals calibrate mucosal immune thresholds and influence the balance between regulatory and inflammatory adaptive responses (33).

Microbiota-derived metabolites, especially short-chain fatty acids (SCFAs), can regulate immune cell metabolism, epithelial function, and T-cell differentiation. Resident microbiota have been shown to influence Treg and Th17 cell balance, which is critical for maintaining mucosal barrier integrity and immune homeostasis (34). When microbial communities are disrupted, this balance may shift toward inflammatory or allergic responses. For example, in colonic immune homeostasis models, gut microbiota-derived short-chain fatty acids have been shown to regulate the size and function of the colonic regulatory T-cell pool. This provides a defined mucosal context in which microbial metabolites promote immune tolerance, rather than implying that all microbiome effects uniformly apply across autoimmune or allergic disease settings (35, 36).

Microbiome dysbiosis therefore acts as an upstream driver of immune bias. Reduced tolerogenic microbial signals or altered metabolite production may weaken Treg-mediated tolerance, enhance Th17-type inflammation, or favor type 2 sensitization depending on tissue context. This provides a mechanistic link between microbiome disturbance and diseases such as inflammatory bowel disease, systemic autoimmunity, asthma, and allergic disease (37).

### 3.4 Barrier disruption and environmental exposure

Environmental exposures can reshape innate immunity by damaging epithelial barriers and lowering the threshold for immune activation. Pollutants, cigarette smoke, allergens, particulate matter, microbial products, and chemical irritants may disrupt epithelial integrity, promote oxidative stress, and increase antigen penetration. When barrier function is compromised, innate immune cells are exposed to higher antigenic and danger-signal loads, creating conditions for exaggerated or misdirected adaptive responses.

A key consequence of barrier disruption is the release of epithelial alarmins. TSLP, IL-33, and IL-25 are rapidly produced after epithelial stress or injury and can activate ILC2s, condition DCs, and promote type 2 immune polarization (4). This mechanism directly links environmental exposure to allergic sensitization and chronic type 2 inflammation.

Environmental exposures are also relevant beyond allergy. Persistent barrier injury may sustain innate inflammatory tone, alter microbial communities, and promote tissue remodeling. Depending on context, this may increase susceptibility to infection, amplify autoimmune inflammation, or bias mucosal immunity toward allergic disease. Barrier disruption is therefore a major upstream modifier of innate immune signal duration and bias.

### 3.4 Age, immune training history, infection/vaccination exposure, and nutritional modulators

Age and prior immune exposures shape innate immune set-points across the lifespan. Aging is associated with immunosenescence and inflammaging, characterized by impaired immune cell function together with chronic low-grade inflammation. This combination can produce an apparently paradoxical state: baseline inflammation is elevated, but acute antimicrobial responses and adaptive priming may be weakened. Such changes contribute to increased infection susceptibility, reduced vaccine responsiveness, and chronic inflammatory disease risk in older adults (38).

Previous infections and vaccinations can also reprogram innate immunity. BCG vaccination, for example, has been shown to induce trained immunity through effects on myeloid cells and hematopoietic progenitors, supporting the idea that immune training can be imprinted beyond mature circulating cells (39). However, the clinical impact of heterologous protection is context-dependent and should not be generalized without trial-level evidence.

Nutritional status further modifies innate immune competence by influencing redox balance, mitochondrial function, and metabolic substrate availability. These effects are usually modulatory rather than disease-specific, but they may interact with aging, infection history, metabolic disease, and microbiome composition to reshape innate immune thresholds. For this reason, age, immune history, and nutrition are best considered systemic modifiers of immune-state programming rather than isolated risk factors (40).

### 3.6 Linking upstream modulators to disease-specific immune outcomes

The upstream modulators discussed above converge on a common principle: they change the operating state of innate immunity. Genetic defects or weak IFN responses may reduce signal strength and impair host defense. Persistent nucleic-acid sensing, metabolic inflammation, or defective clearance of danger signals may prolong innate activation and promote autoimmune feedback. Barrier disruption and epithelial alarmin release may bias immune responses toward type 2 inflammation.

These altered innate states shape adaptive immunity through the three routes discussed in Section 2: antigen presentation and costimulation, cytokine microenvironment formation, and metabolic–epigenetic programming. Therefore, Sections 4–6 examine three disease outcomes as distinct manifestations of altered innate immune regulation: infection as a consequence of insufficient or ineffective innate signaling, autoimmunity as a consequence of persistent and self-amplifying innate activation, and allergic disease as a consequence of type 2–biased epithelial–innate immune signaling.

## 4 Innate immune insufficiency and infection susceptibility

**Figure 2:**
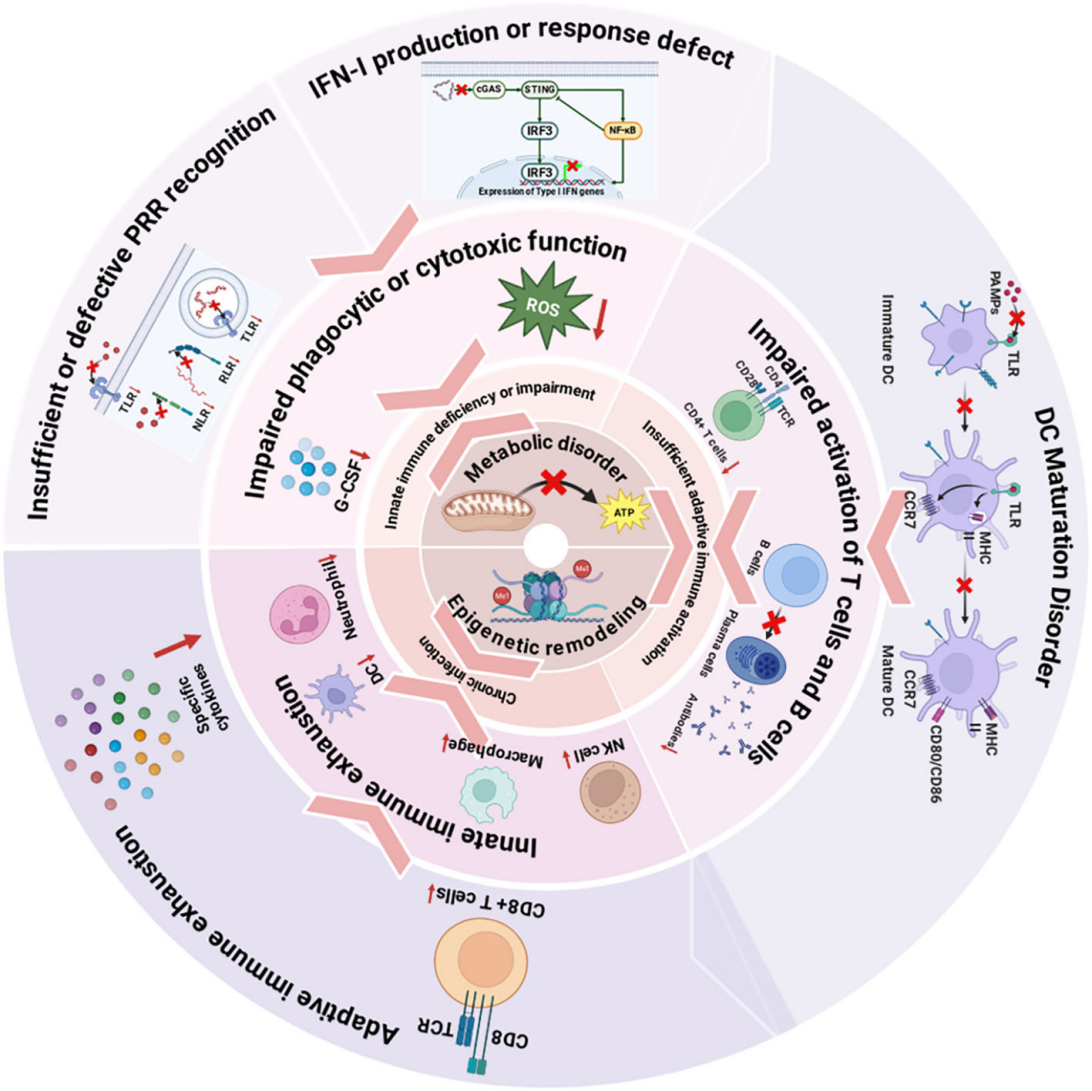
Mechanisms of innate immune dysregulation driving adaptive immune abnormalities and increased susceptibility to infections. This diagram illustrates how dysregulation of innate immunity, through various mechanisms, affects adaptive immune responses and ultimately leads to increased susceptibility to infections. Key mechanisms include insufficient PRR recognition, defects in type I interferon responses, impaired dendritic cell maturation, compromised immune cell functions, adaptive exhaustion and myeloid dysfunction, metabolic disorders, and epigenetic reprogramming.

Infectious susceptibility is a direct consequence of insufficient or ineffective innate immune signaling. In the framework of this review, infection-prone immune dysregulation represents a state in which innate immune signal strength is inadequate, poorly coordinated, or functionally exhausted. This weakens early pathogen containment and compromises the adaptive immune responses required for pathogen clearance and durable protection. Innate immune defects may arise from impaired pathogen sensing, defective interferon responses, reduced phagocytic killing, inadequate DC maturation, or long-term metabolic and epigenetic reprogramming toward immune tolerance. Importantly, infection susceptibility is not caused only by the absence of inflammation. In some settings, especially chronic infection or severe systemic inflammation, excessive early activation may be followed by immune paralysis, reduced antigen presentation, impaired cytokine production, and adaptive immune exhaustion. Therefore, the infection phenotype often reflects a failure to generate appropriately timed and coordinated innate signals rather than simply a globally “weak” immune system (**Figure 2**).

### 4.1 Defective PRR sensing, type I IFN responses, and phagocyte-mediated killing

Early pathogen control depends on rapid detection and elimination of invading microbes. PRRs recognize pathogen-associated molecular patterns and initiate antimicrobial programs, inflammatory cytokine production, and type I interferon responses. When PRR signaling is weak or delayed, the host may fail to generate sufficient early alarm signals, allowing pathogens to replicate and disseminate before adaptive immunity is fully activated (41).

Type I interferons are especially important for antiviral defense. They induce interferon-stimulated genes, regulate infected and neighboring cells, and coordinate innate and adaptive antiviral immunity. Defective activation or regulation of type I interferon responses has been associated with increased severity of viral infections, including severe COVID-19, highlighting the importance of timely IFN-I signaling in early pathogen control (42). Experimental evidence also supports the role of type I IFN receptor signaling in controlling viral replication and organizing innate antiviral responses (43).

Phagocytes provide another essential layer of early defense. Macrophages and neutrophils eliminate pathogens through phagocytosis, oxidative killing, lysosomal degradation, antimicrobial peptides, and cytokine production. When phagocyte function is impaired, pathogens may evade intracellular killing or persist in tissues, increasing the antigenic burden placed on the adaptive immune system. In this setting, adaptive immunity may be activated late, inefficiently, or under inflammatory conditions that favor dysfunction rather than effective memory formation.

In infection-prone states, signal strength can be operationally reflected by inadequate PRR activation, weak IFN-I production, impaired phagocyte killing, reduced DC costimulatory molecule expression, and insufficient cytokine output for T-cell priming. These defects translate into delayed pathogen control, weak CD8^+^ T-cell activation, impaired B-cell help, and poor memory formation.

### 4.2 Impaired DC maturation and defective adaptive priming

DCs are essential for converting innate pathogen sensing into adaptive immune activation. After microbial recognition, DCs mature, upregulate MHC molecules and costimulatory ligands, and produce cytokines that direct T-cell differentiation. If DC maturation is incomplete, adaptive immunity may fail even when pathogens are initially detected. Weak MHC expression, insufficient CD80/CD86/CD40 signaling, or inadequate cytokine production can lead to poor T-cell priming, reduced CD8^+^ cytotoxic responses, impaired Tfh-dependent B-cell help, and suboptimal antibody formation.

Aging provides one example of how altered DC function can weaken adaptive priming. Reviews of human aging indicate that antigen-presenting cell function, including DC function, may be impaired in older individuals, contributing to reduced T-cell responses and weaker immune protection (44, 45). Although the degree of impairment may vary across DC subsets and contexts, the broader principle is clear: reduced antigen presentation and costimulatory output lower the effective strength of the innate signal delivered to adaptive lymphocytes.

Defective DC maturation also affects immune memory. Inadequate inflammatory and costimulatory signals during priming may generate effector responses that are small, poorly differentiated, or short-lived. As a result, subsequent pathogen exposure may not elicit rapid and effective recall responses, further increasing susceptibility to recurrent or persistent infections (46).

### 4.3 Chronic infection, immune exhaustion, and innate-adaptive immune paralysis

Persistent infection or severe systemic inflammation can shift the immune system from activation to suppression. This transition is particularly evident in sepsis and critical illness, where an initial inflammatory phase may be followed by immunosuppression characterized by reduced monocyte HLA-DR expression, impaired cytokine production, lymphocyte dysfunction, and increased risk of secondary infection. Low monocyte HLA-DR expression has been widely studied as a marker of sepsis-associated immunosuppression and has been linked to infection risk and adverse outcomes (47, 48). This interpretation is best supported in the context of sepsis and septic shock. In a longitudinal cohort of septic shock patients, monocyte HLA-DR kinetics were evaluated across different infection sources and pathogens, and delayed or insufficient recovery of mHLA-DR was linked to adverse immune trajectories. Therefore, mHLA-DR should be discussed as a context-specific marker of sepsis-associated immunosuppression and impaired antigen-presenting capacity rather than as a nonspecific indicator of global immune weakness (49).

Immune paralysis illustrates why infection susceptibility cannot be explained solely by insufficient initial activation. In many cases, strong or prolonged innate stimulation leads to compensatory suppression, endotoxin tolerance, or exhaustion-like states in myeloid cells. These cells may show reduced TNF and IL-1β production, decreased antigen presentation, impaired antimicrobial activity, and altered metabolic programs. Reviews of sepsis-induced immunosuppression describe reduced antigen-presenting cell function, endotoxin tolerance, and impaired cytokine production as key contributors to infection vulnerability (50, 51).

This dysfunctional innate state has direct adaptive consequences. Reduced antigen presentation and inflammatory instruction weaken T-cell activation, whereas chronic antigen exposure and persistent inflammatory stress promote T-cell exhaustion. B-cell responses may also become dysregulated, leading to poor antibody quality or ineffective memory. Thus, chronic infection and severe inflammation can produce a combined innate-adaptive failure state (52).

### 4.4 Metabolic and epigenetic tolerance stabilizing hypo-responsive innate states

Innate immune cells can enter durable functional states through metabolic and epigenetic remodeling. While trained immunity enhances responsiveness after prior stimulation, persistent or overwhelming inflammatory stimulation may induce the opposite state: immune tolerance or hypo-responsiveness. In this state, innate cells show reduced inflammatory cytokine production, impaired antigen presentation, and weakened effector functions despite ongoing microbial or inflammatory stimulation (53–55).

Metabolic pathways are closely linked to these functional states. Glycolysis, mitochondrial respiration, TCA-cycle intermediates, and lipid metabolism influence the availability of substrates and cofactors for chromatin-modifying enzymes. As a result, metabolic stress can reshape chromatin accessibility and transcriptional competence, stabilizing either trained or tolerized innate immune programs. Reviews of innate immune memory emphasize that both trained immunity and tolerance involve metabolic and epigenetic adaptations in innate immune cells (56, 57).

In infection-prone states, metabolic–epigenetic tolerance may reduce the ability of monocytes, macrophages, or DCs to respond to secondary microbial challenge. This lowers cytokine output, weakens costimulation, and impairs adaptive priming. Therefore, metabolic and epigenetic remodeling provide a mechanism by which transient infection or inflammation can produce prolonged immune vulnerability.

## 5 Aberrant innate immune activation and autoimmunity

**Figure 3:**
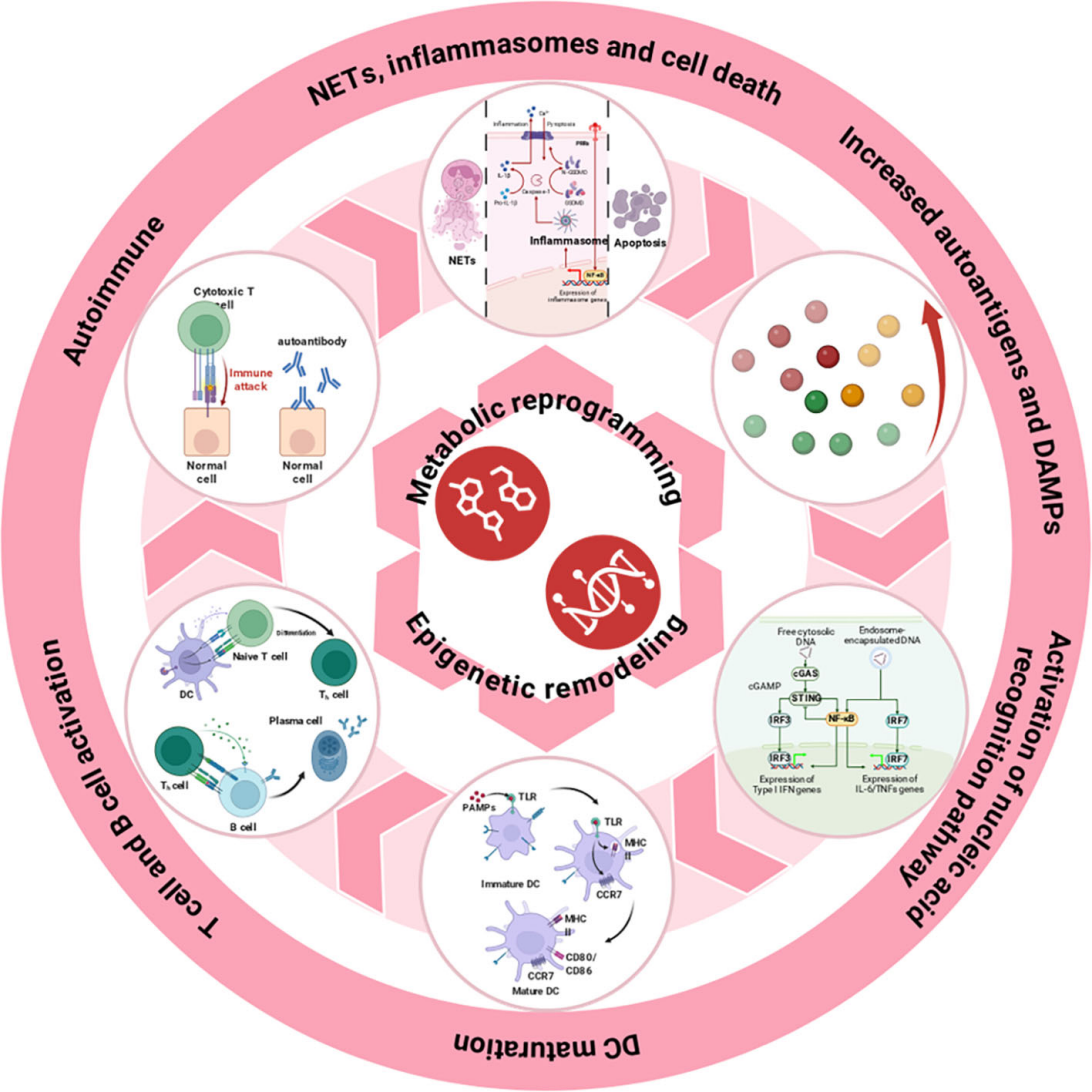
Positive-feedback circuitry by which aberrant innate activation sustains autoimmunity. The circular schematic summarizes major pathogenic nodes in autoimmune disease. The outer ring (clockwise) depicts: (1) increased self-antigens and DAMPs; (2) activation of nucleic-acid–sensing innate pathways (e.g., endosomal TLR7/9 and cytosolic cGAS–STING); (3) dendritic cell (DC) maturation with enhanced antigen presentation and costimulation; (4) activation of T and B cells with autoantibody production; (5) autoantibody-mediated effector injury that amplifies tissue damage and endogenous ligand release; and (6) NETs, inflammasome activation, and dysregulated cell death expanding the immunostimulatory self-antigen pool to reinforce upstream sensing and inflammation. The central module highlights metabolic reprogramming and epigenetic remodeling as a permissive layer that elevates inflammatory tone and alters activation thresholds, thereby potentiating multiple nodes of the loop and promoting chronicity.

Autoimmunity represents a different form of innate immune dysregulation from infection susceptibility. Whereas infection-prone states often reflect insufficient or ineffective innate signaling, autoimmune disease is frequently driven by persistent, excessive, or misdirected innate activation. In this context, innate immune pathways that normally detect infection or tissue damage become chronically engaged by self-derived ligands, leading to loss of tolerance and sustained adaptive immune activation. The key feature of this process is feed-forward amplification. Self-antigens and damage-associated molecular patterns (DAMPs) activate nucleic-acid sensors and inflammasome pathways; these signals promote type I interferon production, DC maturation, and inflammatory cytokine release; activated T and B cells generate autoantibodies and immune complexes; and tissue injury further increases the availability of self-ligands. Thus, autoimmunity can be interpreted as a state in which innate immune signal duration and amplitude are no longer properly restrained (**Figure 3**).

### 5.1 Self-antigens, DAMPs, and aberrant nucleic-acid sensing

A central initiating mechanism in systemic autoimmunity is the inappropriate sensing of endogenous nucleic acids and DAMPs. During tissue injury, defective clearance, infection-associated damage, or abnormal cell death, self-DNA, self-RNA, mitochondrial DNA, histones, and other intracellular components can become extracellular or endosomal ligands for innate immune receptors. When these molecules are not efficiently cleared or are incorporated into immune complexes, they can activate nucleic-acid–sensing pathways that were originally designed for antiviral defense (58–60).

Endosomal TLR7/8/9 and cytosolic DNA-sensing pathways such as cGAS-STING are particularly relevant in this setting. TLR7 recognizes RNA-containing ligands, whereas TLR9 recognizes DNA-containing ligands; both can contribute to systemic autoimmune inflammation when self-nucleic acids become immunostimulatory. Human genetic evidence strongly supports this concept: a TLR7 gain-of-function variant has been shown to cause systemic lupus erythematosus, demonstrating that amplified nucleic-acid sensing can be sufficient to drive lupus-like autoimmunity (61, 62). This represents direct human genetic evidence rather than a purely associative observation. The TLR7Y264H gain-of-function variant was identified in a child with severe lupus, and functional studies showed enhanced sensing of guanosine-related ligands and lupus-like disease when the variant was introduced experimentally. This supports the conclusion that amplified endosomal RNA sensing can be sufficient to drive systemic autoimmunity (63).

This mechanism illustrates how signal bias contributes to disease. The same nucleic-acid sensing pathways that are protective during viral infection can become pathogenic when activation is persistent, self-directed, or insufficiently regulated. In autoimmunity, innate immune bias is often shifted toward IFN-I–dominant and inflammatory programs, setting the stage for adaptive loss of tolerance.

### 5.2 Type I IFN axis, DC maturation, and autoreactive T/B-cell activation

The type I interferon axis is a major amplification module in systemic autoimmunity. In SLE, ongoing IFN-α production and IFN-stimulated gene expression have long been associated with disease activity and immune dysregulation (64–66). Plasmacytoid dendritic cells are an important source of IFN-I, especially when stimulated by immune complexes containing self-nucleic acids. The disease context should be specified here. In SLE, persistent IFN-I pathway activation has been linked to systemic immune activation, and the clinical efficacy of IFNAR1 blockade provides translational support for the pathogenic relevance of this pathway. In randomized SLE trials, anifrolumab, a monoclonal antibody targeting type I IFN receptor subunit 1, improved clinical outcomes compared with placebo, supporting the interpretation that sustained IFN-I signaling is not only a biomarker but also a therapeutically relevant pathway in at least a subset of SLE patients (67).

IFN-I bridges innate sensing to adaptive autoimmunity by enhancing antigen presentation, DC maturation, and costimulatory signaling. Mature DCs present self-antigens more efficiently and provide stronger activation cues to T cells. In parallel, IFN-I can support B-cell activation, survival, differentiation, and autoantibody production, thereby reinforcing immune complex formation and further activation of nucleic-acid sensors (68).

This creates a self-amplifying circuit: self-nucleic acids activate pDCs and other innate cells; IFN-I promotes DC maturation and adaptive immune activation; autoreactive B cells produce autoantibodies; immune complexes deliver additional self-nucleic acids to TLR-containing compartments; and the loop continues. In terms of the framework proposed in this review, IFN-I autoimmunity reflects both increased signal duration and IFN-biased innate immune output.

In autoimmune settings, signal duration is often reflected by persistent nucleic-acid sensing, sustained IFN-stimulated gene expression, repeated inflammasome activation, and ongoing release of DAMPs or NET-derived autoantigens. These prolonged innate signals maintain DC activation, support autoreactive B-cell differentiation, and reinforce self-directed adaptive immunity.

### 5.3 NETs, inflammasomes, and dysregulated cell death expand the self-antigen pool

Innate effector mechanisms can also expand the availability of self-antigens. Neutrophil extracellular traps (NETs) are composed of chromatin and granular proteins released during specialized neutrophil activation. In systemic autoimmunity, excessive NET formation or defective NET clearance can expose DNA, histones, and modified proteins to the immune system, providing both autoantigens and inflammatory stimuli. Reviews of SLE pathogenesis emphasize that imbalance between NET generation and degradation may perpetuate autoimmunity and aggravate tissue injury (69). This mechanism is particularly supported in SLE-related studies. For example, the extracellular DNA sensors AIM2 and IFI16 have been identified as NET-binding autoantigens in SLE; extracellular AIM2-like receptors can bind NETs and form DNase-resistant nucleoprotein fibers targeted by autoantibodies. This provides a specific mechanistic context linking NET formation, impaired degradation, autoantigen persistence, and autoimmune amplification (70).

Inflammasomes provide another route through which innate activation sustains autoimmune inflammation. The NLRP3 inflammasome responds to diverse danger signals and promotes caspase-1 activation and maturation of IL-1β and IL-18. Sustained inflammasome activity can amplify leukocyte recruitment, cytokine production, and tissue damage, thereby generating more DAMPs and maintaining inflammatory feedback. Recent reviews support a role for NLRP3 inflammasome dysregulation in multiple autoimmune diseases, including SLE, rheumatoid arthritis, and inflammatory bowel disease (71–73).

Dysregulated cell death further connects tissue damage to innate immune activation. Apoptotic clearance defects, pyroptosis, necroptosis, and mitochondrial stress can release nuclear and mitochondrial nucleic acids, which activate TLR9, cGAS-STING, and inflammasome-related pathways. In this way, cell death is not simply a consequence of inflammation; it becomes a source of ligands that sustains innate immune activation and adaptive autoreactivity (74, 75).

### 5.4 Metabolic–epigenetic reprogramming and chronic inflammatory set-points

Autoimmune inflammation is often chronic and relapsing, suggesting that immune cells may acquire stable pro-inflammatory states. Metabolic and epigenetic reprogramming provides one mechanism for this persistence. Similar to trained immunity, repeated or chronic inflammatory stimulation can increase chromatin accessibility at inflammatory loci, alter histone modifications, and rewire glycolysis, mitochondrial metabolism, and lipid pathways. These changes may lower the threshold for innate immune reactivation and amplify cytokine responses (76, 77).

This process is relevant because innate immune cells do not respond to each stimulus as if it were the first encounter. Prior inflammatory exposure can alter subsequent responses through durable transcriptional and metabolic changes. In autoimmunity, such trained immunity-like programs may sustain elevated inflammatory tone even between clinical flares, thereby reducing the threshold for reactivation when new triggers arise. Reviews of trained immunity emphasize that epigenetic and metabolic reprogramming can contribute not only to host protection but also to chronic inflammatory and autoimmune pathology (78–80).

Thus, metabolic–epigenetic reprogramming serves as a permissive layer in autoimmune disease. It does not replace antigen-specific adaptive autoimmunity, but it helps maintain the inflammatory environment in which autoreactive T and B cells are repeatedly activated. Therapeutically, this suggests that targeting cytokines or lymphocytes alone may be insufficient in some patients unless upstream innate immune set-points are also corrected (81).

## 6 Innate immune bias and allergic disease

**Figure 4:**
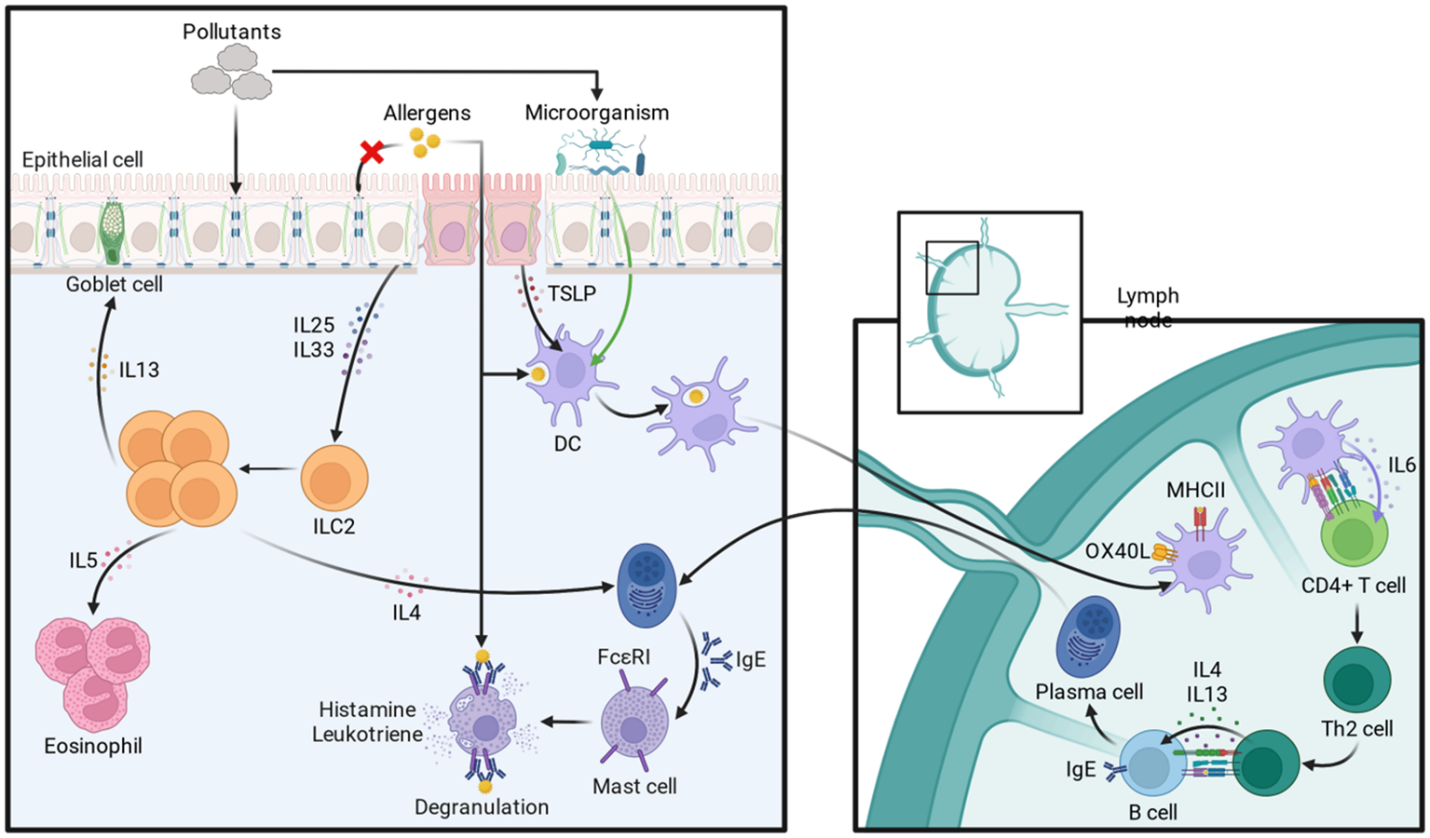
Barrier alarmin–ILC2–type 2 axis and adaptive consequences in allergy. Environmental inputs (pollutants, allergens, and microorganisms) perturb epithelial barriers and induce epithelial-derived mediators (IL-25, IL-33, and TSLP), which initiate coordinated innate and adaptive type 2 programs. Left, IL-25/IL-33 activates ILC2 to produce IL-5 and IL-13, promoting eosinophil recruitment and goblet-cell responses; ILC2-derived IL-4 and related signals facilitate IgE-dependent mast-cell degranulation with release of histamine and leukotrienes. Middle, epithelial signals such as TSLP activate dendritic cells. Right, activated DCs traffic to draining lymph nodes and, via MHC II and costimulatory cues (e.g., OX40L), polarize CD4^+^ T cells toward Th2 and promote B-cell class switching and plasma-cell differentiation to generate IgE. IgE binding to FcϵRI on mast cells establishes an effector amplification loop.

Allergic disease represents a form of innate-adaptive immune dysregulation characterized by qualitative immune bias rather than simple immune deficiency or generalized hyperactivation. In the framework of this review, allergic inflammation is best understood as a type 2–biased innate immune state that instructs adaptive Th2 immunity, IgE production, eosinophilic inflammation, and mast-cell activation. This bias is often initiated at barrier surfaces, where epithelial injury, environmental exposure, and microbial disturbance reshape local innate immune signals. The central pathway linking innate immunity to allergic disease is the barrier–alarmin–ILC2–DC–Th2 axis. Damaged epithelial cells release alarmins such as TSLP, IL-33, and IL-25, which activate ILC2s and condition DCs. ILC2s produce type 2 cytokines including IL-5 and IL-13, whereas conditioned DCs promote Th2 polarization and B-cell IgE class switching. Upon allergen re-exposure, IgE-bound mast cells and basophils amplify allergic symptoms through mediator release. Thus, allergic disease illustrates how innate immune signal bias can determine adaptive immune outcome (**Figure 4**).

### 6.1 Barrier injury and epithelial alarmin release

Epithelial barriers in the skin, airways, and gut are the first sites of contact with allergens, pollutants, microbes, and irritants. Under homeostatic conditions, these barriers limit antigen penetration and support immune tolerance. When epithelial integrity is disrupted, allergens and environmental stimuli penetrate more deeply into tissues and activate local innate immune programs. This shifts the immune environment from tolerance toward sensitization (82, 83).

A major consequence of epithelial injury is the release of alarmin cytokines. TSLP, IL-33, and IL-25 are produced by epithelial cells in response to allergens, pollutants, helminths, viral infection, and mechanical damage. These cytokines act early in allergic inflammation and coordinate both innate and adaptive type 2 responses. Recent reviews emphasize that epithelial alarmins are frontline mediators of allergic responses and play important roles in asthma, allergic rhinitis, atopic dermatitis, and other type 2 inflammatory diseases (84, 85).

Each alarmin contributes to type 2 bias in overlapping but non-identical ways. IL-33 rapidly activates tissue-resident ILC2s and mast cells after epithelial stress; TSLP conditions DCs and promotes Th2-permissive priming; and IL-25 supports ILC2 expansion and type 2 cytokine production. Together, these signals convert barrier damage into a coordinated immune program favoring Th2 inflammation, eosinophil recruitment, mucus production, and IgE-associated effector responses (86).

In allergic disease, signal bias is reflected by epithelial alarmin-dominant output, particularly TSLP, IL-33, and IL-25, which activates ILC2s and conditions DCs to favor Th2 polarization, IgE class switching, and mast-cell-mediated effector responses.

### 6.2 ILC2-mediated amplification of type 2 inflammation

ILC2s are rapid innate effector cells enriched at barrier tissues. They do not require antigen-specific receptors but respond strongly to epithelial alarmins, especially IL-33, IL-25, and TSLP. This allows ILC2s to initiate type 2 inflammation before antigen-specific Th2 responses are fully established. Studies and reviews have highlighted the contribution of ILC2s to asthma, allergic rhinitis, chronic rhinosinusitis, atopic dermatitis, and other allergic diseases (87, 88).

Activated ILC2s produce IL-5 and IL-13, and in some contexts IL-4. IL-5 promotes eosinophil expansion and recruitment, whereas IL-13 contributes to mucus production, goblet-cell differentiation, airway hyperresponsiveness, and tissue remodeling. Through these cytokines, ILC2s amplify the local type 2 environment and create conditions that favor later Th2 polarization and IgE-associated allergic responses.

ILC2 responses also illustrate how innate immune bias can precede adaptive immune polarization. In early allergic inflammation, epithelial alarmins activate ILC2s independently of antigen specificity, generating a cytokine environment that makes Th2 differentiation more likely once DCs present allergen-derived antigens. Thus, ILC2s function as a bridge between barrier-derived danger signals and adaptive type 2 immunity (87, 89).

### 6.3 DC-driven Th2 polarization, IgE class switching, and mast-cell effector amplification

DCs translate epithelial and innate signals into antigen-specific adaptive allergic immunity. After barrier disruption, DCs capture allergens and migrate to draining lymph nodes, where they present antigen to naïve CD4^+^ T cells. In a type 2–skewed epithelial environment, DCs are conditioned to promote Th2 differentiation rather than Th1 or regulatory responses (90).

TSLP is particularly important in this process. TSLP-activated DCs upregulate OX40 ligand (OX40L), reduce IL-12-associated Th1 instruction, and promote inflammatory Th2 differentiation. A landmark study demonstrated that OX40L on TSLP-activated DCs triggers Th2 cell polarization in the absence of IL-12 (91). Additional work has further supported the TSLP–OX40L pathway as a key molecular link between epithelial activation and Th2 immunity (92). A direct experimental context comes from human DC–T-cell coculture systems, in which TSLP-activated dendritic cells were shown to express OX40L but not IL-12, and OX40L on these DCs was required to trigger naïve CD4^+^ T cells to produce Th2 cytokines. This provides a mechanistic example of how epithelial-derived innate signals are converted into antigen-specific adaptive Th2 responses (91).

Once Th2 cells are established, IL-4 and IL-13 promote B-cell class-switch recombination toward IgE. Allergen-specific IgE binds FcϵRI on mast cells and basophils. Upon re-exposure to allergen, cross-linking of IgE-FcϵRI complexes triggers mast-cell degranulation and release of histamine, leukotrienes, prostaglandins, and cytokines, producing acute allergic symptoms and reinforcing local inflammation. Thus, DC-driven Th2 polarization converts innate epithelial alarm signals into antigen-specific adaptive allergic memory and effector amplification (85).

### 6.4 Microbiome dysbiosis and environmental exposure as amplifiers of allergic immune bias

Microbiome composition and environmental exposure modify allergic risk by shaping barrier function and innate immune tone. Early-life microbial exposure can influence immune education, mucosal tolerance, and later susceptibility to allergic disease. Dysbiosis may reduce tolerogenic signals, alter microbial metabolites, impair epithelial integrity, and favor inflammatory or type 2–skewed immune environments. Reviews of asthma and allergy have linked microbial dysbiosis to altered immune development and allergic disease risk (93, 94).

Environmental pollutants, cigarette smoke, particulate matter, and chemical irritants can further amplify allergic bias by injuring epithelial barriers and increasing alarmin release. These exposures may also interact with microbiome dysbiosis, creating a reinforcing circuit: barrier injury changes microbial communities, dysbiosis weakens immune tolerance, and both processes enhance epithelial–innate type 2 signaling.

This section therefore links allergic disease back to the upstream modulators described in Section 3. In allergy, genetic predisposition, barrier fragility, microbiome dysbiosis, and environmental exposure converge to bias innate immunity toward alarmin-dominant and ILC2-activating programs. The adaptive consequence is Th2 polarization, IgE production, and mast-cell-mediated effector amplification.

## 7 Integrated model, therapeutic implications, and future directions

### 7.1 Integrated model: innate immune signal strength, duration, and bias determine adaptive immune outcomes

The sections above support a unified model in which innate immunity regulates adaptive immune fate through three interrelated but operationally distinct dimensions: signal strength, signal duration, and signal bias. These dimensions are not intended as abstract descriptors alone; rather, they can be interpreted through measurable molecular and cellular features of innate immune signaling, including pathway activation amplitude, signaling kinetics, cytokine concentration, antigen-presenting capacity, and the dominant cytokine module generated in a given tissue context.

Signal strength refers to the magnitude of innate immune activation. Operationally, it can be reflected by the intensity of PRR-driven NF-κB or IRF activation, the level of type I interferons and inflammatory cytokines, the expression of MHC molecules and costimulatory ligands such as CD80/CD86/CD40, and the antimicrobial activity of phagocytes. When these outputs are insufficient, DC maturation and T-cell priming are weakened, leading to poor CD8^+^ T-cell responses, impaired Tfh-dependent B-cell help, reduced antibody quality, and increased infection susceptibility. Conversely, excessive signal strength may amplify inflammatory cytokine production and tissue injury, particularly when regulatory feedback is inadequate.

Signal duration refers to the temporal pattern of innate immune activation. Acute and self-limited PRR, IFN-I, or inflammasome signaling may promote pathogen clearance and immune memory, whereas sustained activation can reshape adaptive immunity in a pathogenic direction. For example, persistent IFN-stimulated gene expression and chronic nucleic-acid sensing can maintain DC activation and B-cell stimulation in systemic autoimmunity, while prolonged antigen exposure and inflammatory stress during chronic infection can contribute to T-cell exhaustion and myeloid immune paralysis. Thus, duration captures whether innate signaling is resolved after immune challenge or becomes a self-amplifying inflammatory program.

Signal bias describes the dominant qualitative output of innate immune signaling. Different innate cytokine modules instruct distinct adaptive immune fates: IL-12 and IFN-γ favor Th1 responses; IL-1β, IL-6, IL-23, and TGF-β support Th17 differentiation; TGF-β and IL-2 promote regulatory T-cell development; and epithelial alarmins such as TSLP, IL-33, and IL-25 promote ILC2 activation, Th2 polarization, IgE class switching, and allergic inflammation. In this sense, disease outcomes depend not only on how much innate immunity is activated or how long it persists, but also on which signaling module dominates the innate-to-adaptive immune interface.

Together, these three dimensions explain why innate immune dysregulation can produce divergent disease phenotypes. Insufficient signal strength is most closely associated with infection susceptibility, persistent signal duration with autoimmunity or immune exhaustion depending on context, and type 2-biased epithelial–innate signaling with allergic disease (**Table 3**).

**Table 3:** Operational interpretation of innate immune signal strength, duration, and bias.

Regulatory dimension	Operational indicators	Representative signaling processes	Adaptive immune consequences	Disease relevance
Signal strength	PRR activation amplitude; NF-κB/IRF activation level; IFN-I, TNF, IL-6, and IL-12 concentrations; MHC-II/CD80/CD86/CD40 expression; phagocyte antimicrobial activity	TLR, RLR, or cGAS–STING activation intensity; inflammasome output	Determines DC maturation, T-cell priming intensity, CD8^+^ T-cell responses, Tfh/B-cell help, antibody quality, and memory formation	Insufficient strength promotes infection susceptibility; excessive strength may amplify inflammation
Signal duration	Transient vs sustained cytokine production; persistence of IFN-stimulated genes; chronic inflammasome activity; repeated PRR stimulation; long-term chromatin accessibility changes	Acute antiviral IFN-I response vs chronic IFN-I signature; persistent TLR7/9 or cGAS–STING activation; prolonged NLRP3 activation	Resolved signaling supports protection and memory; persistent signaling promotes T-cell exhaustion, loss of tolerance, or chronic inflammation	Chronic infection, sepsis-associated immune paralysis, systemic autoimmunity
Signal bias	Dominant cytokine or pathway module; IFN-I-dominant, IL-12-dominant, IL-1β/IL-6/IL-23-dominant, tolerogenic, or alarmin-dominant output	IFN-I axis; IL-12/IFN-γ module; inflammasome–IL-1β axis; TSLP/IL-33/IL-25–ILC2 axis	Guides Th1, Th17, Treg, Th2, Tfh, B-cell activation, and IgE class switching	IFN-I bias in autoimmunity; Th17 bias in inflammatory disease; type 2 bias in allergy

### 7.2 Shared and disease-specific mechanisms across infection, autoimmunity, and allergy

Infection susceptibility, autoimmunity, and allergic disease share a common upstream logic: altered innate immune states reshape adaptive immune responses. However, each disease context reflects a distinct pattern of signal strength, duration, and bias.

In infection susceptibility, the dominant abnormality is ineffective innate signal strength. Defective PRR sensing, impaired IFN-I responses, reduced phagocyte function, and weak DC maturation lead to poor T- and B-cell priming, inadequate effector responses, and reduced immune memory. In chronic infection or sepsis, excessive early inflammation may be followed by immune paralysis, producing a secondary state of low responsiveness.

In autoimmunity, the dominant abnormality is persistent and self-directed innate activation. Self-nucleic acids, DAMPs, NETs, inflammasome activation, and IFN-I signaling reinforce one another, creating a positive-feedback loop that promotes DC maturation, autoreactive T-cell activation, B-cell differentiation, autoantibody production, and tissue injury.

In allergic disease, the dominant abnormality is type 2 innate immune bias. Barrier injury and environmental exposure trigger epithelial alarmins, which activate ILC2s and condition DCs to promote Th2 differentiation, IgE class switching, eosinophilic inflammation, and mast-cell effector responses.

Thus, these three disease categories are not isolated phenomena but different manifestations of dysregulated innate-to-adaptive immune instruction.

### 7.3 Therapeutic and clinical implications

The framework proposed here has direct therapeutic implications. If disease outcomes depend on innate immune signal strength, duration, and bias, then treatment strategies should aim not only to suppress inflammation globally but also to recalibrate the specific dysregulated innate immune program.

For infection-prone states, therapeutic strategies may focus on restoring effective innate and adaptive priming. In sepsis-associated immunosuppression, monocyte HLA-DR expression and cytokine production have been explored as immune-state biomarkers, and immunostimulatory approaches such as GM-CSF or IFN-γ have been investigated in selected contexts. Although clinical application remains challenging, these examples highlight the principle that infection susceptibility may require immune restoration rather than broad immunosuppression.

For autoimmune disease, targeting persistent IFN-I signaling is a clinically validated strategy. Anifrolumab, a monoclonal antibody blocking type I IFN receptor subunit 1, has shown efficacy in systemic lupus erythematosus and supports the therapeutic relevance of IFN-I blockade (67, 95). Other innate immune targets, including TLR7/9 signaling, cGAS-STING pathways, NET formation, and inflammasome activity, may also provide opportunities to interrupt autoimmune feed-forward loops, although their therapeutic development requires careful disease- and patient-specific stratification.

For allergic disease, the success of biologics targeting type 2 pathways illustrates the clinical value of correcting innate immune bias. Tezepelumab, an anti-TSLP antibody, reduced exacerbations and improved outcomes in severe uncontrolled asthma, supporting epithelial alarmin blockade as an upstream intervention (96). Therapies targeting IL-4/IL-13 signaling, IL-5, and IgE further demonstrate that the alarmin–ILC2–Th2–IgE axis can be therapeutically interrupted.

Metabolic and microbiome-based interventions represent additional translational opportunities. Because trained immunity and immune tolerance are shaped by metabolic and epigenetic programs, strategies targeting immunometabolism, diet, microbial metabolites, probiotics, postbiotics, or microbiome composition may help modify innate immune set-points. However, such approaches require stronger mechanistic and clinical validation before they can be applied broadly.

### 7.4 Future directions: multi-omics, spatial immunology, early-life programming, and precision medicine

Future studies should move beyond describing isolated immune pathways and instead define how innate immune states are generated, maintained, and translated into adaptive outcomes. Single-cell multi-omics, spatial transcriptomics, proteomics, metabolomics, and longitudinal immune profiling can help identify which innate cell subsets, tissue niches, and molecular programs drive disease-specific adaptive immune responses.

Spatial immunology is particularly important because innate-adaptive interactions are highly tissue dependent. Barrier tissues, lymphoid organs, inflamed lesions, and circulating immune compartments may show distinct immune states even within the same patient. Mapping immune cells together with epithelial, stromal, microbial, and metabolic cues will be essential for understanding why similar innate signals produce different outcomes in different organs.

Early-life immune programming also deserves attention. Microbial exposure, nutrition, infection, vaccination, pollutants, and epithelial barrier development during early life can shape long-term immune tolerance and disease risk. Longitudinal studies linking early-life exposures to later immune signatures may clarify why some individuals develop allergy, autoimmunity, recurrent infection, or immune-mediated comorbidities.

Finally, precision medicine will require immune-state stratification. Patients should not be classified only by clinical diagnosis but also by dominant immune mechanisms, such as IFN-I signatures, inflammasome activity, alarmin-high type 2 inflammation, monocyte HLA-DR suppression, metabolic inflammation, or microbiome dysbiosis. Such stratification may improve therapeutic selection and help explain heterogeneous treatment responses in immune-mediated diseases.
